# Single Genotype of *Anaplasma phagocytophilum* Identified from Ticks, Camargue, France

**DOI:** 10.3201/eid1905.121003

**Published:** 2013-05

**Authors:** Amélie Chastagner, Xavier Bailly, Agnès Leblond, Sophie Pradier, Gwenaël Vourc’h

**Affiliations:** National Center for Biotechnology Information (INRA), Saint Genès Champanelle, France (A. Chastagner, X. Bailly, A. Leblond, G. Vourc’h);; University of Lyon, Marcy l’Etoile, France (A. Leblond);; Ecole Nationale Vétérinaire d’Alfort, Maisons-Alfort, France (S. Pradier);; INRA ENVA ANSES, Maisons-Alfort (S. Pradier)

**Keywords:** Anaplasma phagocytophilum, Ixodes ricinus, Rhipicephalus bursa, Rhipicephalus sanguineus, Rhipicephalus turanicus, Dermacentor marginatus, Hyalomma marginatum, PCR, characterization, ticks, bacteria, tickborne, zoonoses, France

**To the Editor:** Granulocytic anaplasmosis is a tickborne zoonosis caused by *Anaplasma phagocytophilum* bacteria, which are emerging in Europe. Besides infecting humans, *A. phagocytophilum* infect a wide range of wild and domestic mammals (*1*). In Europe, the *Ixodes ricinus* tick is the main vector for the bacteria, but *A*. *phagocytophilum* has also been detected in association with *Rhipicephalus* and *Dermacentor* spp. ticks (*2*). The climate and biotopes of the Mediterranean region are particularly favorable for several species of ticks and, therefore, for tickborne diseases.

Although *I. ricinus* ticks are rare or absent in the Mediterranean Basin, serosurveys performed on equine populations in Camargue, southern France, indicated an *A. phagocytophilum* seroprevalence of ≈10% (*3*). To investigate the prevalence and diversity of *A. phagocytophilum* bacteria in ticks in Camargue, we collected questing ticks from horse pastures and feeding ticks from horses.

Ticks feeding on horses were collected in randomly selected stables during 2007 (84 stables), 2008 (72 stable), and 2010 (19 stables). The stables were chosen among those where evidence of *A. phagocytophilum* seroconversion in horses had been previously found (*3*). In 2008 and 2010, questing ticks were collected by the dragging method in 19 pastures, around bushes, and in areas where horses spent the most time. Surveys were conducted in the spring, which represents the peak activity time of *Ixodes* ticks.

A total of 406 adult ticks were collected, representing 6 species: *Rhipicephalus bursa, R. sanguineus*, *R. turanicus*, *R. pusillus*, *Dermacentor marginatus*, and *Hyalomma marginatum*. Tick species were identified by morphologic criteria and molecular analyses based on mitochondrial 12S rDNA sequences (*4*). Total DNA was extracted from the ticks by using the NucleoSpin Tissue Kit (Macherey-Nagel, Düren, Germany) (*5*). *A. phagocytophilum* was detected by nested PCR targeting the 16S rDNA (Technical Appendix 1).

Of the 406 ticks, 40 were infected with *A. phagocytophilum*. The infected group included ticks from all 6 collected species except *R. pusillus*. Infection rates among the species ranged from 0 to 22% (Technical Appendix 2). The prevalence of *A. phagocytophilum* infection did not differ significantly between species (logistic regression model, p = 0.76) but was higher among questing ticks than feeding ticks (p<0.001; odds ratio 1.15).

We amplified 6 loci by nested PCR (Technical Appendix 1) to characterize *A. phagocytophilum* genetic diversity in positive samples: *ankA*, *msp4*, *pleD*, t*ypA*, and intergenic regions *hemE*–*APH_0021* and *APH_1099*–*APH_1100* (National Center for Biotechnology Information annotation). The GenBank accession numbers for the nucleotide sequences are JX197073–JX197368. No polymorphism was found among the 6 loci tested in the 40 *A. phagocytophilum*–positive ticks. The genotype identified was 100% identical to the reference sequence (NC_007797) for loci *msp4*, *pleD*, and *typA* and for intergenic regions *hemE*–*APH_0021* and *APH_1099*–*APH_1100*. The *ankA* sequence was 96% similar (487 nt) to the reference sequence. The relevance of these loci as markers of diversity was verified (Technical Appendix 3).

To study the phylogenetic relationships between cognate sequences, we included in our analysis all sequences available in GenBank for genes *ankA* and *msp4*. To account for recombination events that affect *ankA* and *msp4* (data not shown) in phylogenetic analyses, we used Neighbor-Net networks (Figure). Phylogenetic analysis of *msp4* (Figure, panel A) indicated that the genotype of *A. phagocytophilum* from ticks in Camargue was included in a clade that also includes genotypes that infect humans and horses in the United States.

**Figure:**
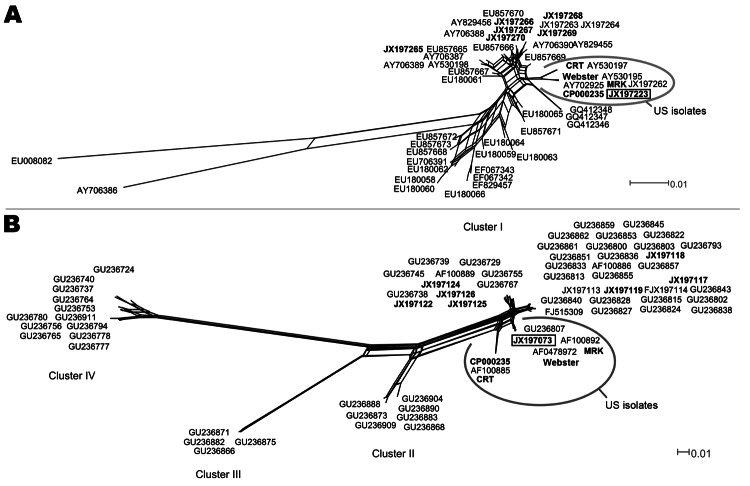
Phylogenetic networks of *Anaplasma phagocytophilum* based on *msp4* (A) and *ankA* (B) genes and built with SplitsTree4 (version 4.11.3; http://splitstree.org/) by the Neighbor-Net method. The sequences of the genotype described in Camargue, France, is framed. Sequences found in the 2 networks are in **boldface**: *A. phagocytophilum* amplified from ticks collected in Combrailles, Auvergne region, France (JX197116–JX197126 and JX197265–JX197270), a human isolate (strain Webster, EU857674 and GU236811]), an American roe deer isolate (strain CRT, JX197261 and JX197113), and *an American horse isolate (*strain MRK, AY530196 and AF153716). Scale bars indicate number of nucleotide substitutions per site.

The diversity of *ankA* sequences has been described as 4 phylogenetic clusters (*6*). All sequences obtained in our study were included in cluster I, particularly in a branch composed exclusively of sequences of *A. phagocytophilum* isolated from humans in the United States (Figure, panel B).

Previous studies investigating *A. phagocytophilum* have revealed a genetic diversity that is thought to have been caused by sympatric epidemiologic cycles involving different vectors and reservoir hosts (*1*,*6*,*7*). In 5 species of ticks (40 ticks total) that we collected from a 250-km^2^ area in southern France, we found only 1 genotype of *A. phagocytophilum*, which we determined to be phylogenetically close to genotypes found in the United States. Sequences phylogenetically related to bacteria in the United States were also observed in Sardinia (*8*) and Sicily (*9*).

The low diversity we found could be explained by a recent introduction of the bacteria into the area [although *A. phagocytophilum*–seropositive horses have been found in the area since 2001 (*3*)] or by a selective sweep linked to the particular ticks and host reservoir in Camargue. The 5 species of ticks that we found positive for *A. phagocytophilum* have been described as potential vectors of *A. phagocytophilum* in the Mediterranean Basin (*2*,*10*). Among the tick species in our investigation, *R. bursa* and *R. sanguineus* ticks are the 2 main carriers of *A. phagocytophilum*, and these ticks are likely to feed on humans and, thus, pose a risk of infection to the local population. Further studies are needed to address the potential effect of *A. phagocytophilum*–infected ticks on human health in this area and, more specifically, the relationship between genotype and pathogenicity.

Technical Appendix 1 Primers used for sequencing *Anaplasma phagocytophilum*.

Technical Appendix 2 *Anaplasma phagocytophilum*–infected ticks collected in Camargue, France, 2007–2010.

Technical Appendix 3 Verification of the nucleotide diversity of *Anaplasma phagocytophilum*, as calculated by use of the Watterson estimator.
