# Supplementary material for: Single Genotype of Anaplasma phagocytophilum Identified from Ticks, Camargue, France
Source: Emerg Infect Dis. 2013 May;19(5):825–6. doi: 10.3201/eid1905.121003 (PMC3647497; doi:10.3201/eid1905.121003)
Supplement: Technical Appendix 1 — Primers used for sequencing Anaplasma phagocytophilum. [file 12-1003-Techapp-s1.pdf]

Article DOI: http://dx.doi.org/10.3201/eid1905.121003

Single Genotype of *Anaplasma phagocytophilum* Identified from Ticks, Camargue, France

Technical Appendix 1

Primers used for sequencing *Anaplasma phagocytophilum*. The primers were defined with the Primer-BLAST software.

Primer		Sequences (5′–3′)	Annealing temperature	Fragment length (bp)
*16S_ external **	*ge3a*	CACATGCAAGTCGAACGGATTATTC	58°C	932
	ge10r	TTCCGTTAAGAAGGATCTAATCTCC		
*16S_ internal **	*ge9f*	ACGGATTATTCTTTATAGCTTGCT	58°C	544
	ge2r	GCAGTATTAAAAGCAGCTCCAGG		
ankA - external	forward	TGAGCCTCACCCGCAGCATG	72°C	540
	reverse	CTCTGCGTTGCTGGAGCCCC		
ankA - internal	forward	CTCACCCGCAGCATGTTG	66°C	534
	reverse	GTTGCTGGAGCCCCTTTATCC		
msp 4 - external	forward	TCGCTGCAATACGATTCCGA	66°C	1300
	reverse	GAGTCTTCCACACCATCGGTT		
msp 4 - internal†	forward	TTAATTGAAAGCAAATCTTGCTCCTATG	66°C	849
	reverse	ATGAATTACAGAGAATTGCTTGTAGG		
pleD - external	forward	ACAAGTGGCCCTGAAGCAAT	66°C	1101
	reverse	TGCGTCGTAGCCTGTCTGCA		
pleD - internal	forward	TGCACTTTGCCGGAGATGGGT	69°C	576
	reverse	same of *pleD*-external reverse		
typA - external	forward	CCTGGACATGCTGACTTCGG	66°C	1455
	reverse	CGGCGGAACTAACCTCACAG		
typA - internal	forward	TGCCTCTGAGGGCCCTATGCC	71°C	550
	reverse	AGCCCTTTCCAGCCCTGCAAC		
hemE-aph0021 - external	forward	GCGATCCTGCCAAGGGTATT	66°C	1070
	reverse	AGCCCTAATTTCCGACCTTGC		
hemE-aph0021 - internal	forward	AGCGCTGTGTGCTTCTTCTGGT	66°C	537
	reverse	AGAGACGCGCTTTCCAGCGA		
aph1099-aph1100 - external	forward	ACAGTGCCCAACCTAGACGA	66°C	1453
	reverse	TGGAAGAACACGGTGGTTGC		
aph1099-aph1100 - internal	forward	GTTGCACATCCTGCTGGGGTGT	69°C	574
	reverse	GCCCCTCTGCAGACAAAGAAGC		

*Published by Massung RF, Slater K, Owens JH, Nicholson WL, Mather TN, Solberg VB, et al. Nested PCR assay for detection of granulocytic ehrlichiae. Journal of Clinical Microbiology. 1998;36:1090-5.
†Published by de La Fuente J, Massung RF, Wong SJ, Chu FK, Lutz H, Meli M, et al. Sequence analysis of the msp4 gene of *Anaplasma phagocytophilum* strains. Journal of Clinical Microbiology. 2005;43:1309-17.

All fragments were amplified by nested PCR. For the first PCR, each reaction contained 40 ng of total DNA in a solution of 25 µL with 1 U of Taq polymerase (Qiagen), 2 µL of each primer at 10 µM, 2 µL of dNTP at 25 mM, 5 µL of Q solution (Qiagen) and 1 µL of $MgCl_2$ at 25 mM. Tests were performed to choose optimal annealing temperatures. The PCR program began by an initial denaturation step of 3 min at 95°C, then 40 cycles consisted of a denaturing step of

30 s at 94°C, an annealing step of 30 s at the temperature corresponding to the target gene (see Table 2) and an extension step of 90 s at 72°C, and finally an extension step of 10 min at 72°C. The nested PCR was performed with 5 µLof the first PCR product in a total volume of 50 µL containing 2 U of Taq polymerase, 4 µL of each primer at 10 µM and 4 µL of dNTP at 25 mM. Nested cycling conditions were as described for the primary amplification.
