# Supplementary material for: Single Genotype of Anaplasma phagocytophilum Identified from Ticks, Camargue, France
Source: Emerg Infect Dis. 2013 May;19(5):825–6. doi: 10.3201/eid1905.121003 (PMC3647497; doi:10.3201/eid1905.121003)
Supplement: Technical Appendix 2 — Anaplasma phagocytophilum–infected ticks collected in Camargue, France, 2007–2010. [file 12-1003-Techapp-s2.pdf]

Article DOI: http://dx.doi.org/10.3201/eid1905.121003

# Single Genotype of *Anaplasma phagocytophilum* Identified from Ticks, Camargue, France

## Technical Appendix 2

Number (%) of collected ticks positive for *Anaplasma phagocytophilum*, Camargue, France, 2007–2010

| | No. positive/no. analyzed (%positive) | | |
|---|---|---|---|
| Genus spp. | Ticks questing in pastures | Ticks feeding on horses | Total |
| *Rhipicephalus bursa* | 3/12 (25) | 19/247 (7.7) | 22/259 (8.5) |
| *Rhipicephalus sanguineus* | 11/53 (20.7) | 2/58 (3.4) | 13/111 (11.7) |
| *Rhipicephalus turanicus* | 2/4 (50) | 0/5 (0) | 2/9 (22.2) |
| *Rhipicephalus pusillus* | 0/9 (0) | 0/1 (0) | 0/10 (0) |
| *Dermacentor marginatus* | 1/3 (33.3) | 1/9 (11.1) | 2/12 (16.7) |
| *Hyalomma marginatum* | 0/0 (0) | 1/5 (20) | 1/5 (20) |
