# Supplementary material for: Single Genotype of Anaplasma phagocytophilum Identified from Ticks, Camargue, France
Source: Emerg Infect Dis. 2013 May;19(5):825–6. doi: 10.3201/eid1905.121003 (PMC3647497; doi:10.3201/eid1905.121003)
Supplement: Technical Appendix 3 — Verification of the nucleotide diversity of Anaplasma phagocytophilum, as calculated by use of the Watterson estimator. [file 12-1003-Techapp-s3.pdf]

Article DOI: http://dx.doi.org/10.3201/eid1905.121003

Single Genotype of *Anaplasma phagocytophilum* Identified from Ticks, Camargue, France

Technical Appendix 3

Verification of the nucleotide diversity of *Anaplasma phagocytophilum*, as calculated by use of the Watterson estimator

To verify the relevance of our loci as markers of *A. phagocytophilum* diversity, we compared the nucleotide diversity of *A. phagocytophilum* found in questing ticks collected in Camargue, France, with that found in 6 questing *Ixodes ricinus* ticks collected in the Combrailles (Auvergne, Central France, HALOS 2010 [*1*]); and with the totality of studied sequences that include 2 cognate sequence obtained in diseased horses from France, 2 American human strains (HGE and Webster), and 1 American roe deer strain (CRT).

The nucleotide diversity was calculated with the Watterson estimator:

$$\theta = \mathrm{K} \quad {}_{i=1}^{n-1} \frac{1}{i}$$

with K the proportion of single nucleotide polymorphisms in the sequences. Diversity in Combrailles ticks showed that the markers used in this study were appropriated to describe significant level of diversity (see graph on following page).

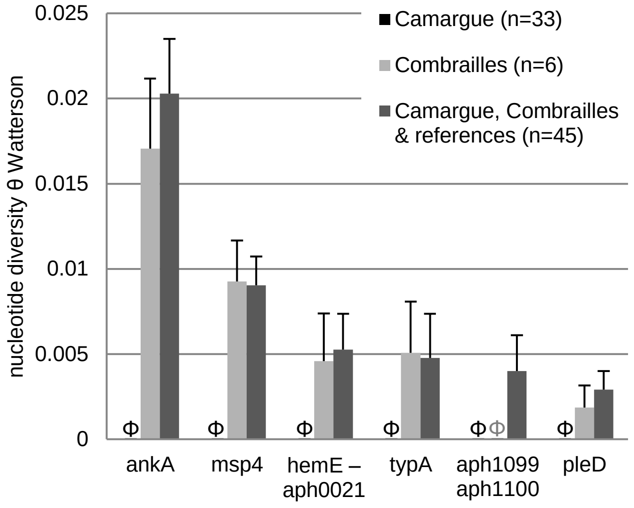

Nucleotide diversity of *Anaplasma phagocytophilum* calculated with the Watterson estimator. n: number of sequences analyzed and Φ indicates the zero values. Error bars indicate upper limit of the standard deviation of the Watterson estimator. Length of the sequences analyzed: 462 pb for ankA, 709 pb for msp4, 457 pb for aph1099-aph1100, 477 pb for hemE-aph0021, 308 pb for typA and 471 pb for pleD.

Reference

1. Halos L, Bord S, Cotte V, Gasqui P, Abrial D, Barnouin J, et al. Ecological factors characterizing the prevalence of bacterial tick-borne pathogens in *Ixodes ricinus* ticks in pastures and Woodlands. Appl Environ Microbiol. 2010;76:4413-20.
